# Development and validation of a gas chromatography method for the determination of β-caryophyllene in clove extract and its application

**DOI:** 10.1038/s41598-021-93306-5

**Published:** 2021-07-05

**Authors:** Mi Hee Park, Chul Jin Kim, Jin Young Lee, In Seon Kim, Sung-Kyu Kim

**Affiliations:** SFC Bio Co., Ltd, 119, Dandae-ro, Dongnam-gu, Cheonan-si, Chungnam, 31116 Korea

**Keywords:** Biological techniques, Planetary science, Health care, Materials science

## Abstract

The purpose of this study is to check the effectiveness of the analysis method that separates and quantifies β-caryophyllene among clove extracts and validate according to current ICH guidelines. The β-caryophyllene was active constituent of clove buds. The developed method gave a good detection response. In the specificity test, the standard solution was detected at about 17.32 min, and the test solution was detected at 17.32 min. The linearity of β-caryophyllen was confirmed, and at this time, the correlation coefficient (R^2^) of the calibration curve showed a high linearity of 0.999 or more in the concentration range. The levels of LOD and LOQ were 1.28 ug/mL and 3.89 ug/mL, respectively. The accuracy was confirmed to be 101.6–102.2% and RSD 0.95 ~ 1.31%. As a result of checking the repeatability and inter-tester reproducibility to confirm the precision, the RSD was found to be 1.34 ~ 2.69%. This validated GC method was successfully applied to a soft capsule containing clove extract and other materials for clinical trials. Therefore, this method can be used as an analytical tool for quality control of various samples, including clove extracts and their products of food and pharmaceutical uses.

## Introduction

The preparation of functional materials and pharmacological products requires the development of good validated methods to ensure its quality, efficacy and safety within different samples. For that, the validation should be completed to confirm that an analytical method is selective, robust, accurate and reproducible at the specified range^1^. To perform the method validation, it is recommended to follow the guidelines on the regulatory agencies^2^. Mandatory Criteria for the quality of natural compounds, pharmaceuticals, foods, cosmetics, and other products are ensured by method validation^3^. Regarding development of therapeutic or functional agents of plans, a validated analytical method is required at every step. Among other things, it is necessary at the step of the quantification of the active compounds in biological components for tracking preclinical and clinical analysis^4^. Moreover, the manufacturing conditions of active pharmaceutical ingredients require proper quality control of the various ingredients involved in the synthesis^5^. The permissible limit of these materials is given in the ICH guidelines^6^.

In this study, we developed and validated analytical method of the estimation of β- caryophyllene in clove extracts to applicates for functional materials according to ICH guidelines. Actually, β-caryophyllene is present in a various plant species such as cloves, cinnamon leaves, copaiba balsam and basil, and a natural bicyclic sesquiterpene^7^ It is a main ingredient in various essential oils obtained from a number of plant species such as the Strobilanthes (~ 7%), Syzygium (~ 13%) and Betula (~ 30%) species^8–10^. It has been known to have antimicrobial, anti-inflammatory, anticancer and antioxidant effects^11–14^. Clove extract oil is produced by extraction from the dried clove buds of the clove plant. Traditionally, it has been applied as a flavouring spice in foods as a fragrance^15,16^. It is also found in topical analgesics. The US Food and Drug Administration (FDA) categorizes clove oil or its components as generally recognized as safe (GRAS) for the purpose of dental cement use or food additive use^17^. In MFDS (Ministry of Food and Drug Safety), the clove buds are classified as a food ingredient. Clove bud is categorized a fragnant plant in vegetable raw materials^18^. Clove represents great potential for pharmaceutical, food, cosmetic and agricultural applications^19^.

Several study has been reported that β-caryophyllene was effective on various diseases including cancer, inflammatory disease and neurodegenerative diseases. β-caryophyllene suppresses ovarian cancer proliferation by inducing apoptosis and cell cycle arrest^20^. β-caryophyllene also induces G1 phase cell cycle arrest in lung cancer cells^21^. β-caryophyllene also induces apoptosis in skin cancer cells including A431 and HaCaT cells by synergistic interaction with aromadendrene oxide 2 and phytol^22^. β-caryophyllene from cloves extract inhibited *H. pylori* growth via via the downregulation of dnaE, dnaN, holB, and gyrA and also downregulated virulence factors such as CagA, VacA, and SecA proteins. Moreover, β-caryophyllene eradicates^23^. *H. pylori* in Mouse Model and the effect was similar with triple therapy^24^. β-caryophyllene also has an effect on inflammatory bowel disease by critical mechanisms^13,25^. β-caryophyllene attenuates DSS-induced colitis, by modulating the expression of genes associated mainly with colon inflammation through inhibition of DSS-induced NF-κB activity. In that experiment, β-caryophyllene reduces the expression of inflammation-related genes including cytokines and chemokines (Ccl2, Ccl7, Ccl11, Ifitm3, IL-1β, IL-28, Tnfrsf1b, Tnfrsf12a); acute-phase proteins (S100a8, Saa3, Hp); adhesion molecules (Cd14, Cd55, Cd68, Mmp3, Mmp10, Sema6b, Sema7a, Anax13); and signal regulatory proteins induced by DSS. Moreover, oral administration of β-caryophyllene significantly reduced the inflammation of colon and reversed the increase in MPO activity and level of IL-6 protein in the tissue^13,25^. β-caryophyllene is also effective on neurodegenerative diseases. β-caryophyllene exerts protective antioxidant effects through the activation of NQO1 in the MPTP model of Parkinson's disease^26^. Trans-caryophyllene also inhibits amyloid β (Aβ) oligomer-induced neuroinflammation in BV-2 microglial cells^27^. Moreover, β-Caryophyllene ameliorates the Alzheimer-like phenotype in APP/PS1 Mice through CB2 receptor activation and the PPARγ pathway^28^.

So, we will apply this material for therapeutic uses. For that, we developed the validation of methods to ensure its quality, safety and efficacy in this work. The method was effectively validated according to the contents: linearity, limit of detection and quantification, selectivity, precision and accuracy. The application of the developed method of commercial formulations containing clove extract will be very satisfactory.

## Results

### Method validation

The validation of the optimized method was performed in accordance with the ICH guidelines. The following parameters were considered: specificity, Linearity, Limit of detection (LOD), limit of quantification (LOQ), Accuracy, Recovery and Precision.

#### Specificity

**Figure 1:**
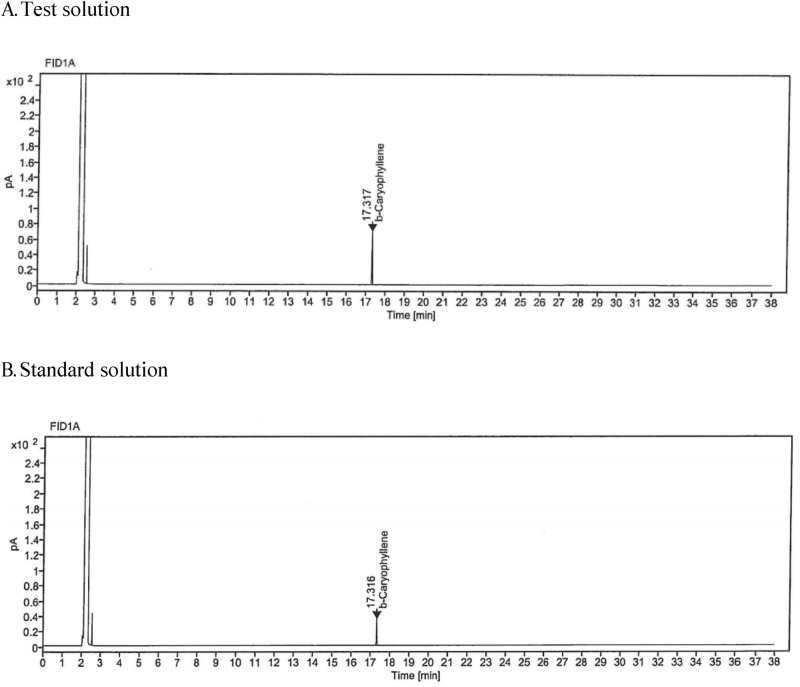
β-caryophyllene chromatogram of test solution (A) and standard solution (B) . The chromatogram data represents the β-caryophyllene in clove extracts (test solution) and standard solution ((-)-trans-Caryophyllene (CAS: 87–44-5 MW: 204.35 g/mol). β-caryophyllene was detected at about 17.32 min in test solution, and at 17.32 min in the standard solution.

The β-caryophyllene of test solution and standard solution was detected at about 17.32 min, and that of test solution was detected at 17.32 min. The chromatograms of the standard solution and test solution are as shown in Fig. 1.

#### Linearity, limit of detection (LOD) and limit of quantification (LOQ)

As for the standard solution, 25 mg of the standard was dissolved in ethanol in a 25 mL diaphragm flask, and the diaphragm was sequentially diluted with ethanol, and samples of 5.04 to 201.6 ug/mL were analyzed by the instrument. As a result, the linearity of β-caryophyllen was confirmed, and at this time, the correlation coefficient (R^2^) of the calibration curve showed a high linearity of 0.999 or more in the concentration range (Table 1 and Supplementary data1). The detection and quantification limit is the value obtained by multiplying the standard deviation of the y-intercept by 3.3 times the standard deviation of the y-intercept by the average value of the slope using the calibration curve analyzed three times as the detection limit, and quantifying the value divided by the average value of the slope of the value multiplied by 10 times. It was set as the limit. As a result, the levels were 1.28 ug/mL and 3.89 ug/mL, respectively.

**Table 1:** Linearity, Limit of detection (LOD) and limit of quantification (LOQ).

Concentration (ug/mL)	Regression equation	R^2^	LOD (ug/mL)	LOQ (ug/mL)
5.04–201.6	y = 1.31x + 1.09	0.9996	1.28	3.89

#### Accuracy and recovery

In order to measure the accuracy of β-caryophyllene in the clove extract, the matrix effect was examined through the percentage recovered by spiking/recovery at different concentrations of the sample. A certain amount of the sample was collected according to the standard substance addition method, and the concentration of 100, 200, and 400 ug/mL of standard solution was added and adjusted to 50 mL, diluted appropriately, and the detection concentration was measured. As a result, the recovery rates for each concentration were 101.6%, 101.6%, and 102.2%, and the relative standard deviations were 1.31%, 1.04%, and 0.95% (Table 2).

**Table 2:** Accuracy of β-Caryophyllene.

Treatment	Spiked Conc.(ug/mL)		
	100	200	400
1	1062.9	1195.65	1394.46
2	1082.26	1175.21	1383.17
3	1093.58	1166.55	1406.98
4	1067.14	1178.61	1376.88
Measured mean (mg/g)	1076.47	1179.00	1390.36
%RSD	1.31	1.04	0.95
Recovery mean(%)	101.6	101.6	102.2

#### Precision

##### Repeatability

In order to check the repetition accuracy for the change in the sample amount, samples were taken at 0.1 g, 0.5 g, and 1.0 g, respectively, and measured repeatedly 6 times each. As a result of checking the relative standard deviation (RSD) of each concentration, it was found to be 1.34 ~ 2.69% (Table 3).

**Table 3:** Repeatability of β-Caryophyllene.

Treatment	Sample content (g)		
	0.1 g	0.5 g	1.0 g
1	1026.65	950.69	969.53
2	950.25	953.14	948.17
3	993.06	973.06	981.01
4	996.01	968.8	967.86
5	1018.7	963.47	969.34
6	1005.16	990.47	985.47
Measured mean (mg/g)	998.3	966.61	970.22
%RSD	2.69	1.51	1.34

##### Reproducibility

In order to confirm the reproducibility of the analysis of the content of β-caryophyllene in the clove extract, the analysis was performed with different analysts and analysis dates. As a result of the analysis between experiments, the content of β-caryophyllene was analyzed as an average of 967.23 mg/g, at which time the standard deviation was 6.03 mg/g and the relative standard deviation was 0.62% (Table 4).

**Table 4:** Reproducibility of β-Caryophyllene.

	Test date and analyst			
	2020.10.20., analyst A		2020.10.23., analyst B	
	0.5 g	1.0 g	0.5 g	1.0 g
1	977.33	994.89	967.11	984.61
2	980.53	975.81	969.48	965.45
3	957.2	958.81	947.05	948.28
4	989.85	958.04	979.69	947.89
5	968.44	959.86	958.09	949.45
6	965.05	983.08	955.02	972.41
Average (mg/g)	973.07	971.75	962.74	961.35
%RSD	0.62			

### Real sample analysis

**Figure 2:**
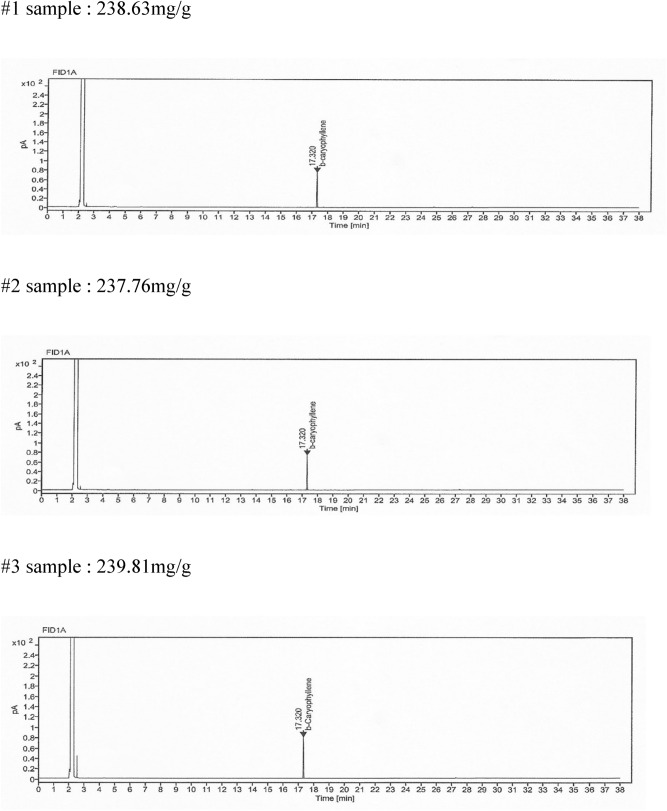
Chromatogram of β-caryophyllene in soft capsule. β-caryophyllene was analyzed the in soft capsule contained clove extract, soybean oil in capsule contents, and modified starch, glycerin, carrageenan, et al.in capsule film. The β-caryophyllene was detected at 17.32 min at a concentration of 238.73 ± 1.02 mg.

The method was applied for real sample determination. We produced the capsules including 243 mg of clove extract (98% β-caryophyllene) and 217 mg of soybean oil in capsule contents, and modified starch, glycerin, carrageenan et al. in capsule film. As shown in Fig. 2, we detected the β-caryophyllene in capsule contents by GC anlysis and the data was 238.73 ± 1.02 mg at 17.32 min. Moreover, we already showed that the β-caryophyllene in medical foods. We inserted 165.3 mg of clove extract (98% β-caryophyllene) for *H.pylori* eradication in the medical foods. After sampling with food, we also detected the β-caryophyllene about 161.25 ± 1.54 mg (data not shown), so we suggested that this method can be useful for quality control.

## Discussion

In this study, a gas chromatography (GC) method was developed and validated for the estimation of major chemical marker, β-caryophyllene in clove extract. GC is the useful analytical technique for essential oil samples. And the ability of the GC method to effectively separate volatiles from a short analysis time is determined by several factors^29,30^. This method has been validated according to ICH guidelines on various parameters, such as specificity, limit of detection, and quantification, linearity, precision and accuracy. The validation parameters tested were found to be within acceptable limits. This method has been successfully applied to the quantification of pharmaceutical formulations.

In this test method, for the determination of β-caryophyllene in the clove extract, a standard substance and a sample were dissolved in ethanol, extracted, and analyzed by GC chromatography. As a result of GC analysis to confirm the specificity, the detection time was detected at 17 min for both the standard solution and the sample, and linearity was confirmed with R^2^ = 0.999 or more in the range of 5.08 to 201.6 ug/mL of the standard solution. As a result of reviewing the recovery rate by adding the standard material, the accuracy was confirmed to be 101.6 ~ 102.2% and RSD 0.95 ~ 1.31%. As a result of checking the repeatability and inter-tester reproducibility to confirm the precision, the average was 978.71 mg/g, and RSD was found to be 1.34 ~ 2.69%.

We applied this method for real sample determination. Actually, we produced the pharmaceutical materials to perform the clinical trials. In the previous study, we showed that the clove extract was effective for the eradication of *H.pylori* in a mouse model. Based on the concentration of animal study, we calculated the human equivalent dose (HED), and then, the daily intake dose was determined. We manufactured the soft capsule to take twice a day, so one capsule contained about 243 mg of clove extract (98% β-caryophyllene). We analyzed the β-caryophyllene concentration of the soft capsule for 3 times, and the β-caryophyllene was detected at 17.32 min at a concentration of 238.73 ± 1.02 mg. Moreover, we already showed that the β-caryophyllene in medical foods. We inserted 165.3 mg of clove extract (98% β-caryophyllene) for *H.pylori* eradication in the medical foods. After sampling with food, we also detected the β-caryophyllene about 161.25 ± 1.54 mg (data not shown). So, this method will be useful for the commercial formulations containing clove extract, especially for applications such as pharmaceutical drugs, functional foods or medical foods.

Other method also estabilished the methods for validation of caryophyllene. However, that method was HPLC method or not an ICH guideline^31,32^. However, GC is the useful analytical technique for essential oil samples. Moreover, there are no standards and test methods for β-caryophyllene for permission of medicines or functional foods yet. Several studies suggested that β-caryophyllene may be useful for therapeutic use for the treatment of cancer, neurodegenerative disease, several inflammatory diseases. So, this method is useful for permission of medicines or functional foods as standards and test methods. We wanted to create internationally accepted standards and analysis methods, and provide useful information to readers who would like to develop pharmaceuticals with caryophyllene in the future.

It is important to point out that β-caryophyllene appear to be the chemical markers for clove extract because a large diversity of studies involving β-caryophyllene have been demonstrated with their high potential as antimicrobial, anti-inflammatory, anti-allergic, insecticidal, and anti-plasmodial. Taking into account the importance of the clove extract for the development of new natural products, and its large use for the functional foods and pharmaceutical and cosmetic industries, as well as the lack of validated analytical methods to accurately quantify these compounds in raw material and its products, we report a complete validated method by gas chromatography.

## Methods

### Instrumentation andanalytical conditions

A gas chromatography with flame ionization detector (Aglient 8890 N(G3540A)) was used for the determination of β-caryophyllene in clove extract. All the gases used in these studies were of pharmacopotential purity. The analysis conditions are as shown below.

**Table:** 

**Item**	Condition
Column	HP-1 (30 m × 0.25 mm, 0.25 um)
Inlet temperature	240℃
Column temperature	50 ℃ (5 min) → 10 ℃/min → 280 ℃ (10 min)
Detector temperature	295℃
Carrier gas and flow	N_2_, 1.0 ml/min
Split ratio	20 : 1
Injection volume	1 ul

### Preparation of clove extract and test solution

Clove extract (98% β-caryophyllene) was obtained from Bordas (Sevilla, Spain). For the preparation of test solution, after weighing a sample, precisely weigh it in a 50 mL volumetric flask, add ethanol to extract it, and filter it with a 0.45 μm nylon filter and use it as a test solution.

### Preparation of standard solution

Take a standard product ((-)-trans-Caryophyllene (CAS: 87–44-5 MW: 204.35 g/mol) and dissolve it in HPLC grade ethanol to prepare it, and dilute it appropriately to use it as a standard solution.

### Production of soft capsule

We prepared the soft capsule for clinical study. The capsule was produced in GMP factory (Suheung, Osong, Korea). We produced the capsules including 243 mg of clove extract (98% β-caryophyllene) and 217 mg of soybean oil in capsule contents, and modified starch, glycerin, carrageenan et al. in capsule film. In this study, we analyzed the β-caryophyllene concentration of the soft capsule.

### Test method

#### Specificity

In order to confirm the specificity, the retention times and degree of separation in the standard and sample were checked. The standard substance and test solution (clove extract containing 98% β-caryophyllene) were analyzed by the same method, and the detected peak was confirmed to confirm that the substance was the same.

#### Linearity, limit of detection (LOD) and limit of quantification (LOQ)

As for the standard solution, 25 mg of the standard was dissolved in ethanol in a 25 mL diaphragm flask, and the diaphragm was sequentially diluted with ethanol, and samples of 5.04 to 201.6 ug/mL were analyzed by instrument. The detection and quantification limit is the value obtained by multiplying the standard deviation of the y-intercept by 3.3 times the standard deviation of the y-intercept by the average value of the slope using the calibration curve analyzed three times as the detection limit, and quantifying the value divided by the average value of the slope of the value multiplied by 10 times.

#### Accuracy, recovery

In order to measure the accuracy of β-caryophyllene in the clove extract, the matrix effect was examined through the percentage recovered by spiking/recovery at different concentrations of the sample. A certain amount of sample was collected according to the standard substance addition method, and the concentration of 100, 200, and 400 ug/mL of standard solution was added and adjusted to 50 mL, diluted appropriately, and the detection concentration was measured.

#### Precision

In order to check the repetition accuracy for the change in the sample amount, samples were taken at 0.1 g, 0.5 g, and 1.0 g, respectively, and measured repeatedly 6 times each. In order to confirm the reproducibility of the analysis of the content of β-caryophyllene in the clove extract, the analysis was performed with different analysts and analysis dates.

## Supplementary Information

Supplementary Information.
