# Supplementary material for: Development and validation of a gas chromatography method for the determination of β-caryophyllene in clove extract and its application
Source: Sci Rep. 2021 Jul 5;11:13853. doi: 10.1038/s41598-021-93306-5 (PMC8257650; doi:10.1038/s41598-021-93306-5)
Supplement: Supplementary file 1 — Supplementary Information. [file 41598_2021_93306_MOESM1_ESM.docx]

**Supplementary data**

Supplementary data 1. Linearity, Limit of detection (LOD) and limit of quantification (LOQ)

| Number of repetitions | 1 | | 2 | | 3 | |
| --- | --- | --- | --- | --- | --- | --- |
|  | Conc. (ug/mL) | Area | Conc. (ug/mL) | Area | Conc. (ug/mL) | Area |
| 1 | 5.04 | 6.905 | 5.04 | 7.161 | 5.04 | 7.632 |
| 2 | 10.08 | 13.936 | 10.08 | 14.044 | 10.08 | 13.998 |
| 3 | 25.2 | 36.226 | 25.2 | 36.418 | 25.2 | 37.072 |
| 4 | 50.4 | 65.874 | 50.4 | 68.683 | 50.4 | 70.121 |
| 5 | 100.8 | 131.287 | 100.8 | 129.593 | 100.8 | 131.312 |
| 6 | 201.6 | 256.655 | 201.6 | 262.917 | 201.6 | 275.587 |
| Inclination | 1.27962 | | 1.29536 | | 1.35371 | |
| y-intercept | 1.40588 | | 1.36925 | | 0.50727 | |
| R^2^ | 0.99979 | | 0.99966 | | 0.99923 | |
| Calibration curve | 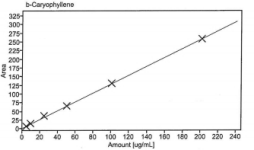 | | 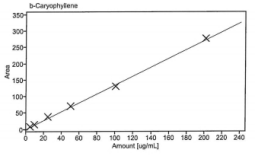 | | 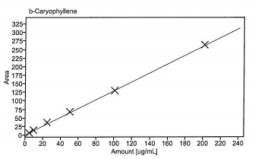 | |
| Slope mean (s) | 1.31 | | SD of y-intercept(δ) | | 0.51 | |
| Limit of detection | 1.28 | |  | |  | |
| Limit of quantification | 3.89 | |  | |  | |
